# Impact of Sleep-Disordered Breathing Treatment on Ventricular Tachycardia in Patients with Heart Failure

**DOI:** 10.3390/jcm11154567

**Published:** 2022-08-05

**Authors:** Muhammed Gerçek, Mustafa Gerçek, Kanjo Alzein, Vanessa Sciacca, Christian Sohns, Philipp Sommer, Volker Rudolph, Henrik Fox

**Affiliations:** 1 Clinic for General and Interventional Cardiology/Angiology, Herz-und Diabeteszentrum NRW, Ruhr-Universität Bochum, 32545 Bad Oeynhausen, Germany; 2 Clinic for Cardiovascular Surgery, Herzzentrum Duisburg, 47137 Duisburg, Germany; 3 Clinic for Electrophysiology, Herz-und Diabeteszentrum NRW, Ruhr-Universität Bochum, 32545 Bad Oeynhausen, Germany; 4 Heart Failure Department, Herz-und Diabeteszentrum NRW, Ruhr-Universität Bochum, 32545 Bad Oeynhausen, Germany; 5 Clinic for Thoracic and Cardiovascular Surgery, Herz-und Diabeteszentrum NRW, Ruhr-Universität Bochum, 32545 Bad Oeynhausen, Germany

**Keywords:** sleep-disordered breathing, implantable cardioverter-defibrillator, ventricular tachycardia, heart failure

## Abstract

Background: Sleep-disordered breathing (SDB) is a highly common comorbidity in patients with heart failure (HF), and a known risk factor for ventricular tachycardia (VT) development. However, little is known about the impact of SDB treatment on VT burden in HF patients to date. Therefore, this study investigated VT burden, as well as implantable cardioverter-defibrillator (ICD) therapies in HF patients with SDB treatment, in comparison to untreated SDB HF patients. Methods: This retrospective study analyzed VT burden, rate of antitachycardia pacing (ATP), and the number of shocks delivered in a propensity score-matched patient cohort of patients with SDB treatment or control. Patients had moderate or severe SDB (n = 73 per each group; standardized mean difference of 0.08) and were followed for a minimum of one year. In addition, survival over 4 years was assessed. Results: Mean patient age was 67.67 ± 10.78 and 67.2 ± 10.10, respectively, with 15.06% and 10.95% of the patients, respectively, being female. Regarding SDB subtypes in the control and SDB treatment group, central sleep apnea was present in 42.46% and 41.09% of the patients, respectively, and obstructive sleep apnea was present in 26.02% and 31.50% of the patients, respectively. Mixed type sleep disorder was present in 31.50% and 27.40% of cases. Among the SDB treatment group, a significantly lower number of VTs (28.8% vs. 68.5%; *p* = 0.01), ATP (21.9% vs. 50.7%; *p* = 0.02), as well as a lower shock rate (5.5% vs. 31.5%; *p* < 0.01), was observed compared to the control group. Furthermore, the VT burden was significantly lower in the SDB treatment group when compared to the time prior to SDB treatment (*p* = 0.02). Event-free survival was significantly higher in the SDB treatment group (Log-rank *p* < 0.01). Conclusion: SDB treatment in HF patients with ICD leads to significant improvements in VT burden, ATP and shock therapy, and may even affect survival. Thus, HF patients should be generously screened for SDB and treated appropriately.

## 1. Introduction

Sleep-disordered breathing (SDB), such as obstructive sleep apnea and central sleep apnea, is very common, but widely underdiagnosed in patients with heart failure (HF), although SDB is strongly associated with increased mortality in HF [1,2,3]. The bidirectional interplay between heart failure and SDB is complex and not well understood. However, SDB, defined as episodes of apnea and hypopnea, has mainly attracted attention as a risk factor for cardiovascular events, hypertension, recurrent sympathetic activation and impairments of cardiac function [4]. SDB was also shown to predispose patients to peri- and postoperative complications, particularly in cardiac surgery, such as myocardial infarction, bleeding, prolonged hospital stay and respiratory complications [5,6].

With regard to cardiac arrhythmia, SDB is known to be an independent risk factor, since SDB affects hemodynamic, autonomic and biochemical alterations as contributors to cardiac rhythm disorders [7]. Accordingly, SDB has been shown to increase the risk of both atrial and ventricular arrhythmia, as well as sudden cardiac death in HF patients [8]. There is growing evidence that the treatment of SDB can mitigate both the occurrence and recrudescence of atrial fibrillation, and supports rhythm stability [9,10]. Patients with symptomatic heart failure suffer from an impaired quality of life and an overall worse outcome, with a well-documented increased risk of ventricular arrhythmia [11]. However, it is still unknown whether SDB treatment has an impact on ventricular arrhythmia, and studies on survival are inconclusive at present [12]. Therefore, it is of the utmost interest to reduce any risk factor for cardiac arrhythmia, not only through establishing lifestyle modifications, but also by controlling contributing factors affecting arrhythmia through specific treatment applications.

Therefore, this study analyzed the impact of SDB treatment on ventricular arrhythmia burden in patients diagnosed with SDB, symptomatic HF, and an implanted cardioverter-defibrillator.

## 2. Patients and Methods

### 2.1. Patient Recruitment

**Figure 1:**
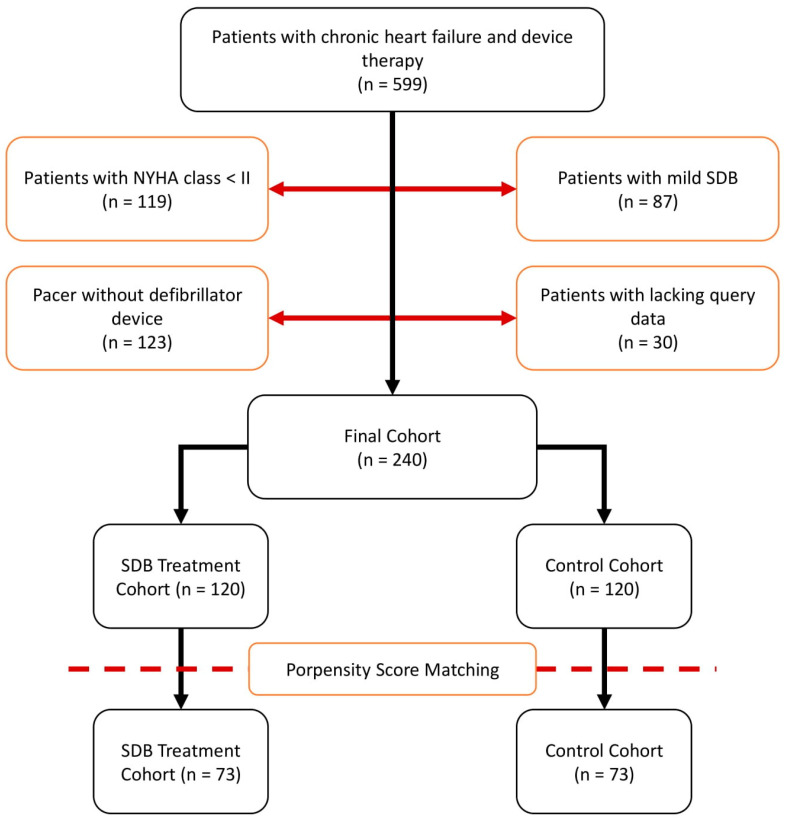
Patient Recruitment and Propensity Score Matching.

Patients hospitalized for symptomatic HF and who received cardioverter defibrillator implantation for both primary or secondary prophylaxis between 1 January 2010 and 31 December 2015, were retrospectively selected, following current guideline recommendations at Herz- und Diabeteszentrum NRW, Bad Oeynhausen, Germany [13]. The main exclusion criteria were the absence of HF symptoms at initial admission (New York Heart Association functional class (NYHA) < II), as well as mild, or no, SDB (apnea–hypopnea index (AHI) < 15). Regarding SDB—after diagnosis—all patients were offered individual SDB treatment according to current recommendations (continuous positive airway pressure (CPAP), Bilevel Positive Airway Pressure (BiPAP), Assisted Spontaneous Breathing (ASB) or phrenic nerve stimulation) [14]. Optimal guideline-derived medical and device therapy had to be established for a minimum of four weeks prior to study eligibility. The study protocol was approved by the institutional ethics committee (2020-588). Patient flow is illustrated in Figure 1.

### 2.2. Multichannel Cardiorespiratory Polygraphy (PG)

To assess sleep-disordered breathing characteristics and to prescribe individual SDB therapy in hospital, overnight multichannel cardiorespiratory polygraphy (Embletta™, Embla, Rotterdam, The Netherlands) was performed at baseline and 1-year follow-up [15]. PG included continuous recording of nasal air flow, chest and abdominal efforts, pulse oximetry, ECG and body positions. Data interpretation was conducted in line with the SERVE-HF study protocol [16], and in accordance with recommendations from the American Academy of Sleep Medicine [17].

Temporary loss of no more than one recording channel, except for nasal airflow, was accepted. Apnea was defined as a ≥90% drop in the flow signal compared to pre-event baseline, for an event duration of at least 10 s. Hypopnea was defined as a drop in nasal pressure signal of 30% from the pre-event baseline, for at least 10 s, associated with a drop of ≥3% in arterial oxygen saturation. SDB severity was defined based on the AHI. Severe SDB was defined as AHI > 30/h, moderate SDB as AHI between 15/h and 30/h, and no, or mild, SDB (nmSDB) as AHI < 15/h. Four SDB categories were used: central sleep apnea (CSA), obstructive sleep apnea (OSA), mixed sleep apnea (mixed SDB) and nmSDB. Respiratory events were defined based on the inspiratory effort: if the entire period of absent airflow was accompanied by continued or increased inspiratory efforts, the apnea was categorized as obstructive. If no inspiratory efforts occurred, apneas were classified as central. Obstructive hypopnea was characterized by a respiratory event meeting the hypopnea criteria, accompanied by either snoring or increased inspiratory flattening of nasal pressure, compared with baseline breathing, or paradoxical thoracoabdominal effort during the event. Central hypopneas were characterized by the absence of flattening of the inspiratory portion of nasal pressure and the absence of paradoxical thoracoabdominal movement. Where these definitions of CSA or OSA did not apply, respiratory events were classified as mixed SDB.

### 2.3. Device Interrogations

ICD or cardiac resynchronization therapy (CRT) devices were programmed and adjusted according to the patient’s requirements. Device interrogations were performed at baseline and 1-year follow-up. Arrhythmias were classified according to 2006 ACC/AHA/HRS key data criteria and definitions for electrophysiological studies and procedures [18]. Recorded events were assessed using the 2014 EHRA/HRS/APHRS expert consensus definitions on ventricular arrhythmias [19]. Additionally, device interrogations were reviewed to collect event-free survival information over 4 years of follow-up.

Assessed parameters included heart rate, atrial pacing (AP), ventricular pacing (VP), biventricular pacing, antitachycardia pacing (ATP) and shock delivery. Ventricular tachycardia was defined as >3 consecutive ventricular extrasystoles with a cycle length of <600 ms and a heart rate of >100/min. Sustained ventricular tachycardia was defined as VT persisting for >30 s, while shorter VT was defined as non-sustained VT.

### 2.4. Statistical Analysis

Statistical analysis was performed using the SPSS-Software (Version 25, IBM Corporation, Armonk, NY, USA). Categorical variables are given as absolute and relative frequencies, and continuous variables as means with standard deviations.

Student’s *t*-test for unpaired and paired parametric samples, or the chi-square test, were performed for group comparisons where appropriate. *p*-values < 0.05 were considered statistically significant. Parameter estimates are provided with their 95% confidence intervals and the corresponding *p*-values.

Since the treatment and control cohorts were analyzed in a non-randomized fashion, we used 1:1 propensity score (PS) matching [20]. To estimate PS, logistic regression models, including all baseline covariates of Table 1 as main effects, were utilized. Good covariate balance was achieved with the main effects model. Matching was performed using SPSS-Software (Version 25, IBM Corporation, Armonk, NY, USA), nearest neighbor matching algorithm without replacement, as recommended by Austin [21]. Good covariate balance and a fair number of matched pairs were achieved with a caliper width of 0.2 standard deviations of the linear predictor [21]. Balance of baseline covariates was assessed by computing the standardized mean differences (<0.2) and *p*-values (>0.05).

## 3. Results

A total of 599 symptomatic chronic heart failure patients with implanted device therapy were retrospectively enrolled. Exclusion criteria applied to 206 patients due to NYHA class < II and/or mild SDB. In addition, 123 patients with pacemakers implanted without defibrillator capability were excluded. Thirty patients had to be excluded for incomplete device interrogation records, resulting in a final study cohort of 240 patients. One hundred and twenty patients received SDB treatment (therapy group), whereas 120 patients refused or could not tolerate SDB treatment (control group). A study flow chart illustrating patient characteristics is depicted in Figure 1.

To achieve group comparability, propensity score (PS) matching was applied. PS matching reduced the overall standardized mean difference from 1.03 to 0.08 in both 73 patient groups (matched pairs), indicating sufficient matching. Before matching, significant differences in either body weight, height, body mass index, comorbidities, coronary artery disease or heart failure etiology, as well as medication disturbances, could be observed in the study patients. All patients were properly matched using PS matching. All baseline characteristic data before and after PS matching are provided in Table 1.

### 3.1. Treatment of SDB

After PS matching, the control and SDB-treated cohort showed a mean age of 67.67 ± 10.78 and 67.2 ± 10.10, respectively, while 15.06% and 10.95% of the patients, respectively, were female.

**Figure 2:**
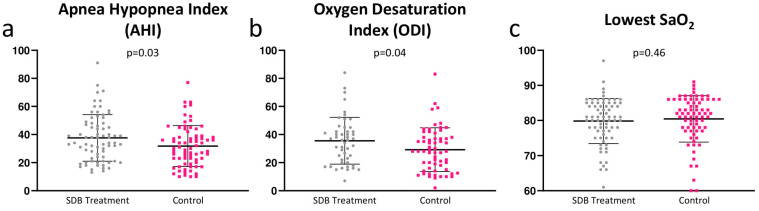
Patients with heart failure and defibrillator therapy presented with a severe sleep-disordered breathing at baseline as expressed in (**a**) Apneo Hyponea Index, (**b**) oxygen desaturation index and (**c**) the lowest oxygen saturation; SDB = sleep disordered breathing.

More severe SDB was present in the treatment group with a higher AHI (31.80 ± 14.55 (control) vs. 37.60 ± 16.56 (treatment), 95%-CI [−10.90; −0.70], *p* = 0.03), oxygen desaturation index (ODI) (15.54 vs. 35.56 ± 16.64, 95%-CI [−12.45; −0.25], *p* = 0.04) and hypopnea duration (52.39 ± 24.38 vs. 67.37 ± 34.09, 95%-CI [−24.85; −5.11], *p* < 0.01) (Figure 2). Regarding SDB subtypes, CSA was comparably present in 42.46% and 41.09%, OSA in 26.02% and 31.50%, and mixed type in 31.50% and 27.40%, respectively (Table 2). The applied SDB treatment was CPAP in 27.4% of the cases, BiPAP in 8.2% of the cases, ASV in 60.3% of the cases, and phrenic nerve stimulation in 4.1% of the cases.

### 3.2. Device Interrogation

**Figure 3:**
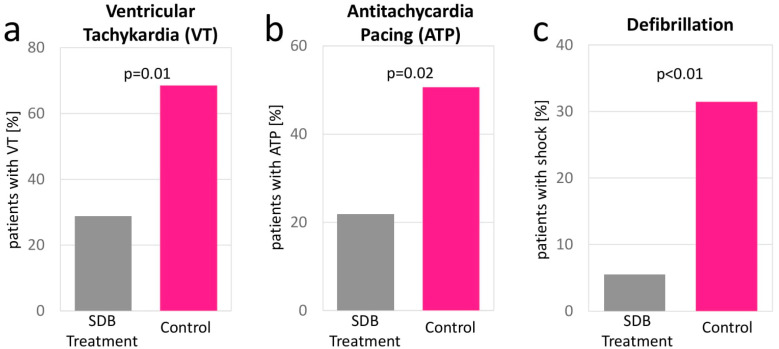
At 1-year follow-up, a significantly lower rate of ventricular tachycardia events (**a**) antitachycardia pacing (**b**) and defibrillation (**c**) was observed in the SDB treatment group.

Device interrogations showed similar occurrences of arrhythmic events, including VT-, ATP- and defibrillation events in both groups at baseline. Ventricular pacing rate was similar at baseline, 65.68 ± 44.86 (control group) vs. 65.75 ± 44.81 (SDB treatment group), 95%-CI [−14.74; 14.60] *p* = 0.99), whereas a higher atrial pacing portion was found in the treatment group (30.05 ± 37.83 vs. 14.80 ± 28.97, 95%-CI [4.17; 26.33] *p* = 0.01). At 1-year follow-up, a significant reduction in total arrhythmic events (32.05 ± 103.25 vs. 6.38 ± 17.77, 95%-CI [1.24; 50.10] *p* = 0.04), VT events (3.78 ± 9.85 vs. 0.50 ± 1.62, 95%-CI [0.94; 5.60] *p* = 0.01), antitachycardia pacing (ATP) events (3.02 ± 9.17 vs. 0.40 ± 1.43, 95%-CI [0.45; 4.78] *p* = 0.02), and defibrillation events (0.53 ± 1.30 vs. 0.05 ± 0.37, 95%-CI [0.16; 0.79] *p* < 0.01) were identified for the SDB treatment group (Figure 3). Details from device interrogations are provided in Table 2. Detailed device interrogation results of the unmatched cohort are provided in Supplementary Table S1.

### 3.3. Changes of Pacer Stimulation and Events

Device interrogation analysis of the control cohort suggests a non-significant tendency (95%-CI strongly tending towards negative values) towards worsening symptoms and an increased need for device therapy, as indicated by an increase in arrhythmic events (95%-CI [−46.92; 1.02], *p* = 0.06), VT events (95%-CI [−4.17; 0.43], *p* = 0.11), ATP events (95%-CI [−3.65; 0.50], *p* = 0.14) and defibrillator events (95%-CI [−0.71; 0.01], *p* = 0.06). In contrast, the SDB treatment group showed a significant decrease in arrhythmic events (95%-CI [5.08; 19.26], *p* < 0.01), VT events (95%-CI [0.40; 4.63], *p* = 0.02), ATP events (95%-CI [0.20; 4.16], *p* = 0.03) and defibrillator events (95%-CI [0.03; 1.04], *p* = 0.04).

**Figure 4:**
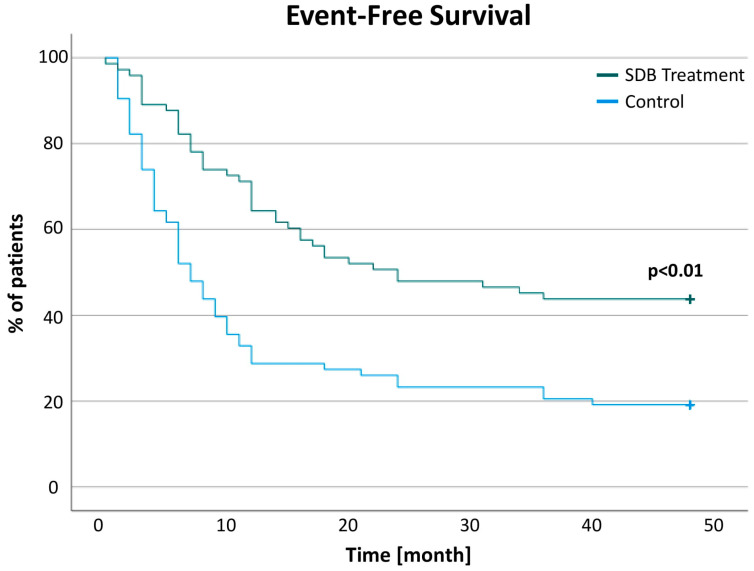
Heart failure patients with ICD and SDB who received SDB treatment showed a significantly longer event-free survival than patients with untreated SDB.

Detailed data of 1-year changes in pacer activity and events are presented in Table 3. Data for the unmatched cohort are available in Supplementary Table S2. Additionally, the SDB treatment group shows a significantly longer event-free survival with a follow-up time of 4 years (Log-rank *p* < 0.01) (Figure 4).

## 4. Discussion

To the best of our knowledge, this is the first study to assess the impact of SDB treatment on ventricular arrhythmia in patients with heart failure monitored by an implanted cardioverter-defibrillator, using a propensity score-matched approach. The key findings are the following: Firstly, the burden of ventricular arrhythmia in patients with SDB and heart failure monitored with implanted cardioverter-defibrillator for both primary or secondary prophylaxis, is high. Secondly, SDB treatment significantly reduced the need for antitachycardia pacing and ICD shock delivery. Thirdly, SDB treatment leads to a longer arrhythmic-free survival. Finally, therefore, patients with heart failure and implanted cardioverter-defibrillator should be widely screened for SDB and receive appropriate SDB treatment.

### 4.1. Arrhythmic Events in Patients with Heart Failure and SDB

Our study confirms that the burden of malignant ventricular arrhythmia is high in patients with heart failure and SDB [22]. Serizawa et al. reported that the presence of SDB was an independent predictor for the occurrence of life-threatening ventricular arrhythmia, and their rate of ICD therapy was higher during sleeping time [23]. Our study showed that 69.5% of patients not receiving SDB treatment suffered from at least one VT event during the 1-year follow-up. Considering the enormous consequences for patients in receiving defibrillator shock therapy, with a negative impact on prognosis and quality of life after each shock [24], it is of the utmost interest to reduce all possible risk factors in the context of VT, such as SDB.

### 4.2. SDB Treatment Reduces the Total Burden of Ventricular Arrhythmia

Hitherto, reports in the literature were inconsistent with regard to the effect of SDB treatment on ventricular arrhythmia [25,26]. Ryan et al. were one of the first to report CPAP to reduce arrhythmic events in patients with obstructive sleep apnea [27]. However, Craig et al. reported that CPAP does not affect dysrhythmia. Although a randomized controlled trial was used, the sample size was very low and the follow-up time was only 4 weeks [28]. Bitter et al. reported that treatment of the Cheyne–Stokes pattern in CSA can reduce arrhythmic events in heart failure patients treated with adaptive servo-ventilation [29], while treatment has to be carefully evaluated due to the increased risk of cardiovascular mortality in the CSA treatment of heart failure patients with reduced ejection fraction [16].

Taken together, our analysis, strengthened by PS matching, now contributes to the field to clarify inconsistencies in the literature, and shows that, in heart failure patients with ICD, SDB treatment significantly increases the arrhythmia-free survival and reduces the need for antitachycardia pacing and shocks.

### 4.3. Clinical Implications

As shown in this study, SDB is contributing to a high ventricular arrhythmia burden which negatively impacts patients’ prognosis and quality of life through the need for any ICD intervention. Therefore, all patients in need of ICD therapy should be widely screened for SDB and treated as appropriate. However, in the presence of heart failure with reduced ejection fraction and central sleep apnea, SDB treatment options in these patients have to be evaluated carefully as adaptive servo-ventilation showed an increased cardiovascular mortality in the large SERVE-HF trial [16]. Newer therapeutic approaches, such as transvenous phrenic nerve stimulation (TPNS), are showing promising results in a long-term follow-up of 5 years, and received FDA approval in 2017 [30]. TPNS might improve CSA, sleep architecture and daytime sleepiness [31]. However, whether or not there is any beneficial impact on arrhythmic events has yet to be investigated in forthcoming studies.

### 4.4. Limitations

The main limitations of our study were its retrospective, single-center design, as well as the small sample size, and data starting from 2010. Propensity score matching was applied to mimic prospective randomization, such as cohort matching, ensuring the best possible matching of retrospective data, but not replacing the need for further prospective controlled, randomized investigation. Additionally, medication records were not consistent, and thus an antiarrhythmic medication-derived change in arrhythmic burden cannot be fully excluded.

## 5. Conclusions

SDB treatment in patients with symptomatic chronic heart failure leads to a significant reduction in ventricular tachycardia burden and reduces device therapy requirements, demonstrating the known strong connection between SDB and cardiac impairment. SDB treatment may even have an impact on survival, which has to be investigated in larger, prospective, randomized and controlled clinical trials.

## Figures and Tables

**Table 1:** Baseline Characteristics and Propensity Score Matching.

Variable			Before PS-Matching				After PS-Matching			
			Control n = 120	Treated n = 120	SMD	*p*-Value	Control n = 73	Treated n = 73	SMD	*p*-Value
**Baseline Characteristics**	**Baseline Characteristics used for Propensity Score Matching**	**Age [years]**	66.97 ± 11.14	66.72 ± 10.20	−0.02	0.86	67.67 ± 10.78	67.2 ± 10.10	−0.05	0.79
		**Gender_female_ [n (%)]**	21 (17.50)	12 (10.00)	0.25	0.92	11 (15.06)	8 (10.95)	0.14	0.46
		**Height [cm]**	173.70 ± 12.49	175.70 ± 8.20	0.24	0.14	173.73 ± 14.99	175.9 ± 7.92	0.26	0.28
		**Weight [kg]**	85.16 ± 18.00	92.22 ± 18.46	0.38	**<0.01**	87.63 ± 17.61	88.89 ± 18.42	0.07	0.67
		**BMI [kg/m²]**	27.78 ± 4.93	29.89 ± 5.51	0.38	**<0.01**	28.38 ± 4.60	28.57 ± 5.32	0.03	0.82
		**NYHA [class]**	2.74 ± 0.54	2.51 ± 0.70	−0.32	**0.01**	2.63 ± 0.57	2.69 ± 0.57	0.10	0.47
		**II [n (%)]**	28 (25.83)	36 (40.00)	-	-	24 (3288)	20 (27.40)		
		**III [n (%)]**	86 (71.66)	70 (58.33)	-	-	49 (67.12)	52 (71.23)		
		**IV [n (%)]**	3 (2.50)	2 (1.66)	-	-	0 (0.00)	1 (1.37)		
		**Rhythm baseline**	1.93 ± 1.18	1.86 ± 1.24	−0.05	0.67	1.9 ± 1.16	1.91 ± 1.28	0.01	0.95
		**(I) SR [n (%)]**	71 (59.16)	79 (65.83)	-	-	44 (60.27)	47 (64.38)		
		**(II) AFlut [n (%)]**	2 (1.66)	0 (0.00)	-	-	0 (0.00)	0 (0.00)		
		**(III) AFib [n (%)]**	31 (25.83)	19 (15.83)	-	-	21 (28.76)	11 (15.06)		
		**(IV) Pacemaker [n (%)]**	22 (18.33)	16 (13.33)	-	-	8 (10.95)	15 (20.54)		
		**History of CPR [n (%)]**	2 (1.66)	2 (1.66)	0.00	1.00	1 (1.36)	2 (2.73)	0.11	0.56
		**Hypertension [n (%)]**	72 (60.00)	85 (70.83)	0.24	0.08	46 (63.01)	51 (69.86)	0.15	0.38
		**CAD [n (%)]**	86 (71.66)	62 (51.66)	−0.40	**<0.01**	47 (64.38)	49 (67.12)	0.05	0.73
		**DCM [n (%)]**	28 (23.33)	56 (46.66)	0.47	**0.00**	22 (30.13)	23 (31.50)	0.03	0.86
		**HCM [n (%)]**	4 (3.33)	3 (2.50)	−0.05	0.70	1 (1.36)	1 (1.36)	0.00	1.00
		**other CM [n (%)]**	11 (9.16)	6 (5.00)	−0.19	0.21	7 (9.58)	5 (6.84)	−0.13	0.55
		**Diabetes [n (%)]**	42 (35.00)	56 (46.66)	0.23	0.07	24 (32.87)	28 (38.35)	0.11	0.49
		**CKD [n (%)]**	59 (49.16)	64 (53.33)	0.08	0.52	38 (52.05)	42 (57.53)	0.11	0.51
		**CKD [stage]**	1.21 ± 1.35	1.34 ± 1.38	0.09	0.48	1.26 ± 1.32	1.46 ± 1.41	0.15	0.37
		**0 [n (%)]**	62 (51.66)	56 (46.66)	-	-	35 (47.94)	31 (42.46)	-	-
		**1 [n (%)]**	2 (1.66)	3 (2.50)	-	-	2 (2.73)	2 (2.73)	-	-
		**2 [n (%)]**	28 (23.33)	33 (27.5)	-	-	21 (28.76)	21 (28.76)	-	-
		**3 [n (%)]**	24 (20.00)	20 (16.66)	-	-	12 (16.43)	13 (17.80)	-	-
		**4 [n (%)]**	4 (3.33)	8 (6.66)	-	-	3 (4.10)	6 (8.21)	-	-
		**Dialysis [n (%)]**	3 (2.50)	5 (4.16)	0.08	0.47	3 (4.10)	4 (5.47)	0.07	0.70
		**COPD [n (%)]**	10 (8.33)	14 (11.66)	0.10	0.39	9 (12.32)	10 (13.69)	0.04	0.81
		**Device**	2.3 ± 0.89	2.46 ± 0.83	0.20	0.14	2.41 ± 0.85	2.3 ± 0.91	−0.13	0.45
		**(0) no [n (%)]**	2 (1.66)	0 (0.00)	-	-	1 (1.36)	0 (0.00)	-	-
		**(I) 1-chamber ICD [n (%)]**	29 (24.16)	26 (21.66)	-	-	14 (19.17)	22 (30.13)	-	-
		**(II) 2-chamber ICD [n (%)]**	20 (16.66)	12 (10.00)	-	-	12 (16.43)	7 (9.58)	-	-
		**(III) CRT [n (%)]**	69 (57.50)	82 (68.33)	-	-	46 (63.01)	44 (60.27)	-	-
		**ACE/AT1-Blocker [n (%)]**	111 (92.50)	112 (93.33)	0.03	0.80	64 (87.67)	68 (93.15)	0.22	0.26
		**Diuretics [n (%)]**	110 (91.66)	107 (89.16)	−0.08	0.51	65 (89.04)	67 (91.78)	0.09	0.57
		**Beta Blocker [n (%)]**	111 (92.50)	116 (96.66)	0.23	0.15	69 (90.00)	69 (94.52)	0.00	1.00
		**Digitalis [n (%)]**	11 (9.16)	12 (10.00)	0.03	0.83	7 (9.58)	3 (4.10)	−0.18	0.19
		**Amiodarone [n (%)]**	49 (40.83)	42 (35.00)	−0.12	0.35	29 (39.72)	28 (38.35)	−0.03	0.87
		**Sotalol [n (%)]**	4 (3.33)	5 (4.16)	0.04	0.73	3 (4.10)	3 (4.10)	0.00	>0.99
		**other antiarrhythmic medication [n (%)]**	13 (10.83)	6 (5.00)	−0.27	0.09	5 (6.84)	6 (8.21)	0.06	0.75
		**MRA [n (%)]**	84 (70.00)	98 (81.66)	0.30	**0.04**	57 (78.08)	55 (75.34)	−0.07	0.70
		**LVEF [%]**	31.09 ± 9.94	30.81 ± 9.69	−0.03	0.92	30.69 ± 9.94	30.04 ± 9.23	−0.07	0.68
		**SDB**								
		**CSA [n (%)]**	51 (42.50)	47 (39.16)	-	-	31 (42.46)	30 (41.09)	-	-
		**OSA [n (%)]**	33 (27.50)	41 (34.16)	-	-	19 (26.02)	23 (31.50)	-	-
		**Mixed [n (%)]**	36 (30.00)	32 (26.67)	-	-	23 (31.50)	20 (27.40)	-	-

Abbreviations: standardized mean difference (SMD), body mass index (BMI), New York Heart Association classification (NYHA), sinus rhythm (SR), atrial flutter (Aflut), atrial fibrillation (AF), cardiopulmonary resuscitation (CPR), coronary artery disease (CAD), dilated cardiomyopathy (DCM), chronic kidney disease (CKD), chronic obstructive pulmonary disease (COPD), implantable cardioverter-defibrillator (ICD), cardiac resynchronization therapy (CRT), angiotensin-converting-enzyme inhibitors (ACE), angiotensin II receptor type 1 antagonists (AT1-Blocker), mineralocorticoid receptor antagonist (MRA), Sleep-Disordered Breathing (SDB), central sleep apnea (CSA), obstructive sleep apnea (OSA).

**Table 2:** Device Interrogation and Multichannel Cardiorespiratory Polygraphy Analysis of the Control and SDB-Treated Patients in the PS Matched Cohorts.

Variable		PS Matched Cohort			
		Control (n = 73)	Treated (n = 73)	95%-CI	*p*-Value
**Pacemaker**	**HR min (PG) [/s]**	48.61 ± 10.58	55.80 ± 12.85	−11.26; −3.13	**<0.01**
	**HR max (PG) [/s]**	92.04 ± 23.24	89.66 ± 24.39	−5.80; 10.56	0.57
	**HR mean (PG) [/s]**	64.77 ± 9.14	65.23 ± 10.73	−3.90; 2.97	0.79
	**Time to first rhythmic event since Embletta [month]**	8.06 ± 8.63	13.02 ± 10.94	−8.83; −1.09	**0.01**
	**Pacemaker Queries (2010–2014)**	9.45 ± 5.12	11.06 ± 6.15	−3.47; 0.23	0.09
	**Arrhythmic events 1 year before SDB treatment**	8.72 ± 16.51	18.73 ± 42.44	−20.89; 0.85	0.07
	**VT events 1 year before SDB treatment**	1.97 ± 4.56	3.04 ± 8.65	−3.37; 1.21	0.36
	**ATP events 1 year before SDB treatment**	1.50 ± 4.29	2.60 ± 8.21	−3.28; 1.06	0.31
	**Defibrillation events 1 year before SDB treatment**	0.19 ± 0.86	0.59 ± 2.06	−0.94; 0.13	0.14
	**VT events 1 year after SDB treatment**	3.78 ± 9.85	0.50 ± 1.62	0.94; 5.60	**0.01**
	**ATP events 1 year after SDB treatment**	3.02 ± 9.17	0.40 ± 1.43	0.45; 4.78	**0.02**
	**Defibrillation events 1 year after SDB treatment**	0.53 ± 1.30	0.05 ± 0.37	0.16; 0.79	**<0.01**
	**AHI/h**	31.80 ± 14.55	37.60 ± 16.56	−10.90; −0.70	**0.03**
	**ODI/h**	29.22 ± 15.54	35.56 ± 16.64	−12.45; −0.25	**0.04**
	**AI/h**	17.97 ± 14.34	19.78 ± 18.44	−8.10; 4.47	0.57
	**Hypopnea Duration (mean) [s]**	52.39 ± 24.38	67.37 ± 34.09	−24.85; −5.11	**<0.01**
	**Apnea Duration (max) [s]**	42.42 ± 18.65	47.34 ± 24.9	−12.29; 2.45	0.19
	**Mean SaO_2_ (%)**	92.42 ± 2.46	91.76 ± 2.90	−0.23; 1.53	0.14
	**Lowest SaO_2_ (%)**	80.23 ± 7.39	79.13 ± 10.17	−1.82; 4.00	0.46
	**NT-proBNP [pg/mL]**	4712.04 ± 7423.21	3480.93 ± 4448.61	−1598.1; 4060.3	0.39

Abbreviations: heart rate (HR), Multichannel Cardiorespiratory Polygraphy (PG), Sleep-disordered breathing (SDB), ventricular tachycardia (VT), antitachycardia pacing (ATP), apnea–hypopnea index (AHI), oxygen desaturation index (ODI), arousal index (AI), N-terminal prohormone of brain natriuretic peptide (NT-proBNP).

**Table 3:** 1-Year Outcome after SDB Treatment (PS-matched cohort).

PS-Matched Cohort								
Variable	Control				SDB Treatment			
	Baseline	1 Year	95%-CI	*p*-Value	Baseline	1 Year	95%-CI	*p*-Value
**Arrhythmic events**	8.72 ± 16.51	31.66 ± 103.92	−46.92; 1.02	0.06	18.73 ± 42.44	6.56 ± 17.99	5.08; 19.26	**<0.01**
**VT events**	1.97 ± 4.56	3.83 ± 9.91	−4.17; 0.43	0.11	3.04 ± 8.65	0.52 ± 1.64	0.40; 4.63	**0.02**
**ATP events**	1.50 ± 4.29	3.06 ± 9.23	−3.65; 0.50	0.14	2.60 ± 8.21	0.42 ± 1.44	0.20; 4.16	**0.03**
**Defibrillation events**	0.19 ± 0.86	0.54 ± 1.31	−0.71; 0.01	0.06	0.59 ± 2.06	0.05 ± 0.37	0.03; 1.04	**0.04**

Abbreviations: Sleep-Disordered Breathing (SDB), ventricular tachycardia (VT), antitachycardia pacing (ATP).

## Data Availability

The data underlying this article will be shared upon reasonable request to the corresponding author.

## Supplementary Materials

The following supporting information can be downloaded at: https://www.mdpi.com/article/10.3390/jcm11154567/s1, Table S1: Device Interrogation and Multichannel Cardiorespiratory Polygraphy Analysis of the Control and SDB Treated patients in the Unmatched and PS Matched Cohorts; Table S2: 1-year Outcome after SDB Treatment (unmatched cohort).
