# Supplementary material for: Impact of Sleep-Disordered Breathing Treatment on Ventricular Tachycardia in Patients with Heart Failure
Source: J Clin Med. 2022 Aug 5;11(15):4567. doi: 10.3390/jcm11154567 (PMC9369567; doi:10.3390/jcm11154567)
Supplement: Supplementary file 1 [file jcm-11-04567-s001.zip › jcm-1841283-supplementary.pdf]

Supplementary Table S1. Device Interrogation and Multichannel Cardiorespiratory Polygraphy Analysis of the Control and SDB Treated patients in the Unmatched and PS Matched Cohorts.

	Variable	Unmatched Cohort				PS Matched Cohort			
		Control (n = 120)	Treated (n = 120)	95%-CI	p-value	Control (n = 73)	Treated (n = 73)	95%-CI	p-value
Pacemaker	HR min (PSG) [/s]	49.62 ± 10.82	55.47 ± 11.88	−8.91; −2.80	**<0.01**	48.61 ± 10.58	55.80 ± 12.85	−11.26; −3.13	**<0.01**
Pacemaker	HR max (PSG) [/s]	90.30 ± 23.64	91.53 ± 24.55	−7.70; 5.21	0.71	92.04 ± 23.24	89.66 ± 24.39	−5.80; 10.56	0.57
Pacemaker	HR mean (PSG) [/s]	64.50 ± 9.69	65.14 ± 10.36	−3.34; 2.04	0.64	64.77 ± 9.14	65.23 ± 10.73	−3.90; 2.97	0.79
Pacemaker	Time to first rhythmic event since Embletta [month]	8.09 ± 7.93	13.32 ± 11.59	−8.41; −2.07	**<0.01**	8.06 ± 8.63	13.02 ± 10.94	−8.83; −1.09	**0.01**
Pacemaker	Pacemaker Queries (2010–2014)	9.80 ± 4.91	10.95 ± 6.74	−2.66; 0.35	0.13	9.45 ± 5.12	11.06 ± 6.15	−3.47; 0.23	0.09
Pacemaker	Arrhythmic events 1 year before SDB treatment	8.07 ± 15.12	33.26 ± 200.79	−62.38; 12.00	0.18	8.72 ± 16.51	18.73 ± 42.44	−20.89; 0.85	0.07
Pacemaker	VT events 1 year before SDB treatment	1.91 ± 4.80	2.53 ± 8.71	−2.43; 1.19	0.50	1.97 ± 4.56	3.04 ± 8.65	−3.37; 1.21	0.36
Pacemaker	ATP events 1 year before SDB treatment	1.64 ± 4.85	2.25 ± 8.46	−2.39; 1.16	0.50	1.50 ± 4.29	2.60 ± 8.21	−3.28; 1.06	0.31
Pacemaker	Defibrillation events 1 year before SDB treatment	0.30 ± 1.15	0.41 ± 1.65	−0.48; 0.25	0.55	0.19 ± 0.86	0.59 ± 2.06	−0.94; 0.13	0.14
Pacemaker	VT events 1 year after SDB treatment	3.35 ± 8.52	0.42 ± 1.32	1.37; 4.49	**<0.01**	3.78 ± 9.85	0.50 ± 1.62	0.94; 5.60	**0.01**
Pacemaker	ATP events 1 year after SDB treatment	2.40 ± 7.46	0.34 ± 1.16	0.68; 3.41	**<0.01**	3.02 ± 9.17	0.40 ± 1.43	0.45; 4.78	**0.02**
Pacemaker	Defibrillation events 1 year after SDB treatment	0.50 ± 1.32	0.06 ± 0.36	0.19; 0.68	**<0.01**	0.53 ± 1.30	0.05 ± 0.37	0.16; 0.79	**<0.01**
Pacemaker	AHI/h	30.18 ± 15.09	36.06 ± 15.44	−9.77; −2.00	**<0.01**	31.80 ± 14.55	37.60 ± 16.56	−10.90; −0.70	**0.03**
Pacemaker	ODI/h	28.32 ± 15.93	33.56 ± 15.84	−9.95; −0.53	**0.03**	29.22 ± 15.54	35.56 ± 16.64	−12.45; −0.25	**0.04**
Pacemaker	AI/h	16.72 ± 14.51	16.35 ± 16.68	−4.35; 5.06	0.88	17.97 ± 14.34	19.78 ± 18.44	−8.10; 4.47	0.57
Pacemaker	Hypopnea Duration (mean) [sec]	52.12 ± 25.36	65.07 ± 30.76	−20.41; −5.50	**<0.01**	52.39 ± 24.38	67.37 ± 34.09	−24.85; −5.11	**<0.01**
Pacemaker	Apnea Duration (max) [sec]	41.11 ± 21.50	44.78 ± 24.25	−9.65; 2.29	0.23	42.42 ± 18.65	47.34 ± 24.9	−12.29; 2.45	0.19
Pacemaker	Mean SaO2 (%)	92.41 ± 2.40	92.05 ± 2.56	−0.28; 0.99	0.27	92.42 ± 2.46	91.76 ± 2.90	−0.23; 1.53	0.14
Pacemaker	Lowest SaO2 (%)	80.86 ± 7.77	79.40 ± 8.83	−0.66; 3.57	0.18	80.23 ± 7.39	79.13 ± 10.17	−1.82; 4.00	0.46
	NT-proBNP [pg/ml]	4143.13 ± 6196.41	2767.20 ± 3649.00	−365.11; 3116.96	0.12	4712.04 ± 7423.21	3480.93 ± 4448.61	−1598.14; 4060.34	0.39

Supplementary Table S2. 1-year Outcome after SDB Treatment (unmatched cohort).

Unmatched Cohort								
**Variable**	**Control (n = 120)**				**SDB Treatment (n = 120)**			
	**Baseline**	**1 year**	**95%-CI**	**p-value**	**Baseline**	**1 year**	**95%-CI**	**p-value**
**Arrhythmic events**	8.07 ± 15.12	22.83 ± 82.13	−29.48; −0.05	**0.05**	33.26 ± 200.79	5.78 ± 15.32	−9.47; 64.42	0.14
**VT events**	1.91 ± 4.80	3.41 ± 8.58	−3.08; 0.07	0.06	2.53 ± 8.71	0.43 ± 1.34	0.46; 3.72	**0.01**
**ATP events**	1.64 ± 4.85	2.44 ± 7.52	−2.15; 0.55	0.24	2.25 ± 8.46	0.35 ± 1.17	0.32; 3.46	**0.02**
**Defibrillation events**	0.30 ± 1.15	0.51 ± 1.33	−0.53; 0.10	0.19	0.41 ± 1.65	0.06 ± 0.36	0.03; 0.66	**0.03**
